# Outcome of Fascia Lata Frontalis Sling Suspension in a Challenging Case of Post-traumatic Aponeurotic Ptosis with an Upgaze Limitation: A Case Report

**DOI:** 10.7759/cureus.108256

**Published:** 2026-05-04

**Authors:** Mansi Pandey, Anupam Singh, Madhubari Vathulya, Barun Kumar

**Affiliations:** 1 Ophthalmology, All India Institute of Medical Sciences, Rishikesh, Rishikesh, IND; 2 Plastic and Reconstructive Surgery, All India Institute of Medical Sciences, Rishikesh, Rishikesh, IND; 3 Cardiology, All India Institute of Medical Sciences, Rishikesh, Rishikesh, IND

**Keywords:** aponeurotic ptosis, crawford technique, fascia lata, frontalis suspension surgery, trauma

## Abstract

Ptosis is drooping of the upper lid margin in primary gaze, which can be broadly classified as congenital and acquired. Acquired ptosis is further classified as myogenic, aponeurotic, neurological, mechanical, and traumatic. Ptosis secondary to trauma is caused by heterogeneous mechanisms. It is usually associated with distorted anatomy, making the management challenging and outcomes unpredictable. We present the case of a 46-year-old male patient, diagnosed with right eye complete post-traumatic aponeurotic ptosis with fair Bell's and poor levator palpebrae superioris action, vertical dystopia, upgaze limitation, and upper lid scarring. He had difficulty in pursuing his occupation and maintaining a livelihood as complete ptosis led to the loss of binocularity and depth perception, which also led him to a high risk of occupational hazards. Therefore, we planned the patient's frontalis suspension procedure using autogenous fascia lata and the Crawford technique based on the risks and possible outcomes explained. Postoperatively, the patient's marginal reflex distance 1 (MRD 1) was 2 mm. The patient did not have any diplopia, achieved binocularity, and was satisfied with the results. He is managing well his day-to-day activities without any difficulty with depth perception.

## Introduction

Ptosis can be broadly classified as congenital and acquired based on the time of onset. It can also be classified into aponeurotic, myogenic, neurogenic, and mechanical types based on the mechanism of causation [1,2]. The most common type of acquired ptosis is the senile, followed by post-traumatic [3]. There are various treatment options available for the management of ptosis, but the deciding factor is the severity of ptosis, the amount of levator palpebrae superioris (LPS) action, Bell's phenomenon, and the condition of the ocular surface. The standard of care includes surgical interventions, such as Muller muscle resection, LPS advancement, resection, and frontalis suspension surgeries. The optimal surgical correction should not pose a risk to vision, should eliminate the need for reoperations, and should result in patient satisfaction [4-6].

We report a successful outcome of frontalis sling surgery using autologous fascia lata, performed with the Crawford technique, in a challenging case of post-traumatic severe aponeurotic ptosis. The case was complicated by fair Bell’s phenomenon, poor LPS function, vertical dystopia, limitation of upgaze, and significant upper eyelid scarring with adhesions.

## Case presentation

**Figure 1:**
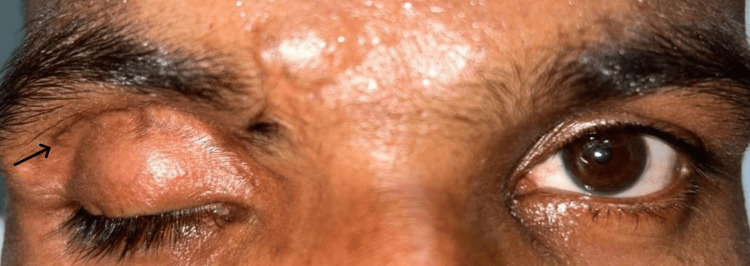
Clinical photograph of the right eye showing complete ptosis with a scar mark near the superior orbital margin (black arrow).

A 46-year-old male presented with complete dropping of the right eyelid after trauma with a metallic sheet for three years. His best corrected visual acuity (BCVA) by Snellen’s chart was 6/9 (P) in the right eye (RE) and 6/6 in the left eye (LE), and near vision was N6 in both eyes by reduced Snellen chart at 33cm with cycloplegic refraction of -1.5DS of RE and -0.5 x 90◦ of LE. Pupils were normal in shape and normally reacting. There was complete ptosis of the RE with a scar mark present near the superior orbital rim (Figure 1, black arrow) with an absent lid crease. Also, a vertical orbital dystopia of 2 mm was noted. The ocular surface evaluation was done using Schirmer’s test (total), which was 10 mm, and tear film breakup time, which was 5 seconds in the RE. Slit lamp evaluation of the anterior segment of the RE revealed a few superficial punctate keratopathies (SPKS), which were present inferiorly, and the rest of the findings were within normal limits. No abnormality was detected on the dilated posterior segment examination of the RE. All the anterior and posterior segment findings were within normal limits in the LE.

The margin reflex distance 1 (MRD 1) was -4 mm in the RE and 4 mm in the LE, pointing towards RE severe ptosis; the rest of the parameters of ptosis evaluation are shown in Table 1.

**Table 1:** Preoperative ptosis work-up details of the patient. MRD: margin reflex distance; LPS: levator palpebrae superioris

Measured Parameters	RE	LE
MRD 1	-4 mm	4 mm
MRD 2	6 mm	5 mm
MRD 3	2 mm	7 mm
Horizontal Palpebral Length	28 mm	28 mm
Vertical Palpebral Height	2 mm	9 mm
Extraocular Movement	-2 of Upgaze Limitation Present, Rest Full and Free in All Gazes	Full and Free in All Gazes
Margin Crease Distance	Could Not Be Measured Due to Absent Lid Crease	6 mm
Bell's Phenomenon	Fair	Good
Marcus Gunn Jaw Winking	-	-
Ice Pack Test	-	-
LPS Action (Berke method)	3 mm (poor)	12 mm (good)

**Figure 2:**
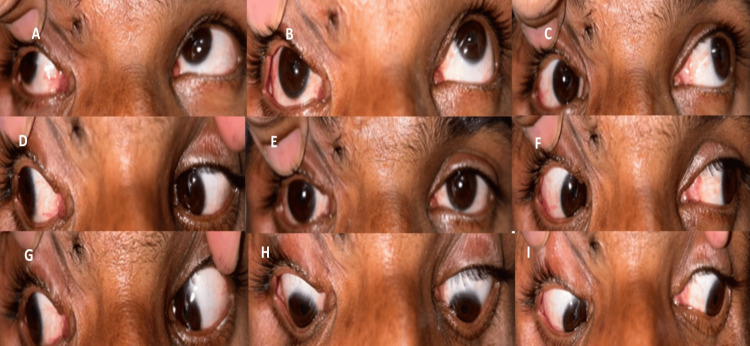
Preoperative nine gaze photographs (A-I) showing an upgaze limitation (A-C) in the right eye.

Thus, a provisional diagnosis of RE upper lid post-traumatic severe (complete) ptosis with fair bells and poor LPS action with vertical dystopia with an upgaze limitation (Figure 2) with an adherent scar at the superior orbital rim was made.

**Figure 3:**
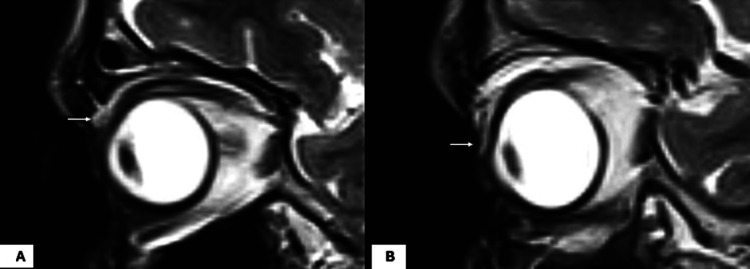
T2-weighted oblique-sagittal section of MRI orbit showing disinserted and retracted LPS aponeurosis in the right eye (white arrow in A) and the aponeurosis inserted into the upper border of the tarsal plate (white arrow in B) in the left eye.

The patient was subjected to various investigations, including contrast-enhanced magnetic resonance imaging of the brain and orbit to rule out any other sequelae of trauma, which revealed disinsertion and retraction of LPS aponeurosis up to the superior orbital rim from a tarsal plate of the RE (site of insertion) (Figure 3A, white arrow), whereas it was normal on the left side (Figure 3B, white arrow).

**Figure 4:**
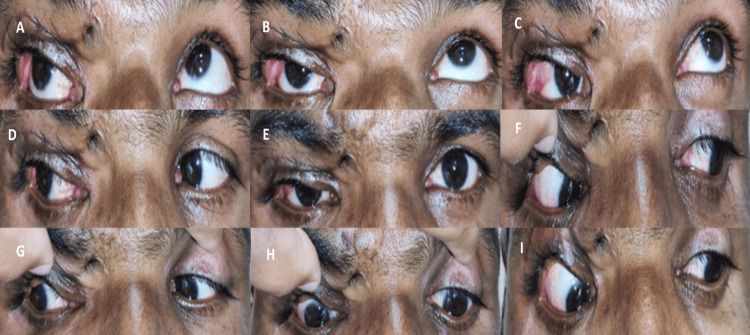
Postoperative nine gaze photographs (A-I) showing no obscuration of visual axis in the primary gaze (E) showing a satisfactory postoperative outcome.

Thus, following investigations, we reached a final diagnosis of RE upper lid post-traumatic aponeurotic complete ptosis with fair Bell’s and poor LPS action, along with vertical dystopia with an upgaze limitation with an upper lid adherent scar. After thorough evaluation, the patient was planned for RE ptosis correction with some degree of under-correction in view of the compromised Bell’s and upgaze limitation with frontalis sling using autogenous fascia lata by the Crawford technique under general anesthesia. Surgery was significantly challenging due to adherent scar tissue and local tissue distortion, but it was successfully completed with the combined efforts of the ophthalmic and plastic surgery teams. Postoperatively, the patient was administered a short course of oral and topical antibiotics, along with a long course of eyedrop carboxymethylcellulose 0.5% twice hourly and eye ointment hydroxypropyl methylcellulose 0.5% twice hourly for a week, then six times per day to mitigate the risk of postoperative exposure keratopathy. The donor site of fascia lata was healthy on subsequent follow-up visit. After one year of follow-up, his BCVA in the RE was 6/9, and there was no obscuration of the visual axis with MRD1 being 2mm; he was having only a few SPKs inferiorly on anterior segment examination by slit lamp as in the preoperative period, but the patient was asymptomatic, joined his profession, and was satisfied without any evidence of adverse outcomes (Figure 4).

## Discussion

Ptosis is defined as an abnormally low-lying upper eyelid margin in the primary gaze. Acquired ptosis is classified into various categories depending on the etiology, including aponeurotic, myogenic, neurogenic, traumatic, and mechanical. Trauma is said to be the second most common cause of acquired ptosis in some studies [7,8]. Trauma can lead to ptosis by different mechanisms, including direct and indirect injury to eyelid elevators. Direct injury includes direct trauma to the muscle, either LPS or Muller’s muscle, while indirect causes include penetrating neck injuries, nerve compression by intraorbital foreign bodies, etc. [3]. This heterogeneous nature of traumatic ptosis makes its management difficult and prognosis unpredictable, but the line of management remains the same as with other kinds of ptosis. Ptosis surgery is functionally indicated when there is a significant reduction in upper eyelid height, often taken as an MRD 1 of 2 mm or less, along with a documented loss of the upper visual field on perimetry, and may also be considered when the droop causes notable cosmetic dissatisfaction for the patient.

In a ptosis surgery, both functional success and good cosmetic results are important [9]. Various surgical options are available depending upon the severity of the ptosis and the amount of levator function, which are measured during the thorough workup of the patient. As in the case of mild acquired ptosis with good levator muscle function, surgery can be directed at Müller’s muscle; options for this include Müller’s muscle-conjunctival resection or the Fasanella-Servat procedure [10,11]. If there is dehiscence or disinsertion of the aponeurosis with good levator muscle function, it can be corrected by advancing and reattaching the aponeurosis through either an anterior or posterior route. In cases where levator action is only fair to good (more than 4 mm), a levator resection is preferred, with the extent of resection decided according to the muscle’s preoperative function [11]. In cases of poor (<4 mm) levator function, the desired upper eyelid elevation can be provided via Whitnall’s ligament suspension or frontalis suspension. The posttraumatic scarring and distorted anatomy pose challenges for Whitnall’s ligament suspension; therefore, Frontalis suspension should be preferred in such cases.

Frontalis suspension can be carried out through several sling-passing patterns, such as the single triangle, double triangle (Crawford) [11], single rhomboid (Friedenwald-Guyton) [12], double rhomboid (Iliff), double trapezoid (Wright), single pentagon (Fox), double pentagon, and the Mehta modification of the modified Crawford approach. These techniques can be performed with a variety of sling materials, but the Fox and modified Crawford methods are the most frequently compared. While both offer similar results in terms of appearance, functional improvement, and complication rates, the modified Crawford technique is often favored. It tends to produce less lagophthalmos, gives a more defined and aesthetically pleasing eyelid crease in the immediate post-operative period, addresses lateral droop more effectively, and avoids a visible forehead scar [9]. The only major drawback is that it needs a greater length of suspension material and takes more time to perform compared to the Fox technique.

There have been various kinds of suspension materials, which can be endogenous or exogenous. Endogenous materials include preserved or fresh fascia lata, fascia temporalis, palmaris longus tendon, and umbilical vein [11], while the most commonly used exogenous materials are silicone rod, Mersilene polyester fiber mesh, Supramid monofilament nylon, and Gore-Tex nonabsorbable monofilament suture. Autogenous fascia lata remains the preferred choice overall, as it carries a lower risk of recurrence and fewer postoperative complications compared to other materials. However, in children under 3-4 years of age, obtaining an adequate length of fascia lata is challenging, and concerns about leaving a permanent scar on the thigh often discourage parents. In such cases, surgeons may opt for synthetic materials or banked fascia lata. Some surgeons also choose synthetic options because they are easy to source, require less complex surgical handling, and avoid operating in an unfamiliar anatomical area where harvesting a suitable fascia strip of adequate length and width can be difficult, but the correction may not last for long due to the limited half-life of the synthetic materials [13].

Furthermore, there are many factors that may make the ptosis correction challenging, like compromised Bell’s, upgaze limitation, and dry eyes, leading to SPKs. Our patient had all three conditions in addition to disinserted and retracted LPS and an upper eyelid scar, making the ptosis correction very challenging.

In view of the significantly compromised day-to-day activity of the patient, the procedure was planned in collaboration with the plastic surgery team, keeping the aim of under-correction of the ptosis.

Ptosis surgery can significantly enhance a patient’s ability to carry out daily activities and visual tasks, thereby improving functional performance. However, it is important to first assess whether surgery is truly necessary. For instance, mild cases of traumatic ptosis often recover well without intervention, likely due to the natural tissue tightening that occurs from fibroblast activity during healing [14].

The risks of ptosis correction range from short-term effects such as bruising, bleeding, or infection, to longer-term issues like scarring, irregular eyelid crease formation, asymmetry, and over- or under-correction. Overcorrection in particular may lead to complications such as lagophthalmos and exposure keratopathy. Achieving good symmetry in unilateral ptosis can be especially challenging, as the operated eyelid must match the appearance of the unaffected side.

Ultimately, the decision to proceed with surgery should balance the expected functional and cosmetic gains against the possibility of undesirable outcomes. Careful preoperative measurements combined with precise surgical execution are key to ensuring optimal results and patient satisfaction.

## Conclusions

This case helps us to understand that even in complex post-traumatic aponeurotic complete ptosis with poor levator function, fair Bell’s phenomenon, upgaze limitation, vertical dystopia, and significant upper lid scarring, a carefully planned frontalis sling using autogenous fascia lata can yield a favorable functional and anatomical outcome. Thorough preoperative evaluation, realistic surgical planning with intentional under-correction, and a multidisciplinary approach were crucial in minimizing postoperative exposure-related complications and optimizing visual rehabilitation. Beyond cosmetic improvement, suitable surgical intervention restored the patient’s visual axis, functional binocularity, and occupational ability, significantly enhancing his quality of life. This report underscores the importance of individualized surgical decision-making in traumatic ptosis, where tailored management can achieve predictable and satisfactory long-term results even in challenging scenarios.
